# HPMC-ZnO Nanorods Enhance Hydrophilicity and Contact-Killing Activity on Polypropylene Meshes and Sutures

**DOI:** 10.3390/ph19010055

**Published:** 2025-12-26

**Authors:** Sangita Jana, Akshit Malhotra, Honey Mittal, Sambuddha Chakraborty, Manika Khanuja, Gyanendra Singh, Ram Karan, Elvira Rozhina, Ashwini Chauhan

**Affiliations:** 1 Department of Microbiology, University of Delhi-South Campus, Delhi 110021, India; 2 Department of Microbiology, Tripura University (A Central University), Tripura 799022, India; 3 Centre for Nanoscience and Nanotechnology, Jamia Millia Islamia, Delhi 110025, India; 4 Department of Physics & Environmental Sciences, Sharda School of Engineering & Science, Sharda University, Greater Noida 201306, India; 5 Biological Sciences Division, ICMR-National Institute of Occupational Health, Ahmedabad 380016, India; 6 Institute of Fundamental Medicine and Biology, Kazan Federal University, 420008 Kazan, Russia; rozhinaelvira@gmail.com

**Keywords:** bacterial biofilms, anti-adhesive coatings, hernia mesh, polypropylene sutures, HPMC, zinc oxide nanoparticles, nanotoxicology

## Abstract

**Background:** Biomedical device-associated infections pose major challenges in surgical care, particularly in hernia repair where polypropylene (PP) meshes and sutures are prone to bacterial colonization and biofilm formation. The limitations of antibiotic resistance and toxicity warrants the need of developing innovative antibacterial strategies. **Methods:** We developed a composite coating of hydroxypropyl methylcellulose (HPMC) and zinc oxide nanorods (ZnO NP) synthesized via thermal decomposition. This coating was applied to PP meshes and sutures to enhance anti-adhesive properties. The study evaluated surface hydrophilicity through water contact angles, estimation of Zn^2+^ ions using inductively coupled plasma–mass spectrometry (ICP-MS), and long-term efficacy over six months. Safety was assessed via systemic toxicity studies in murine models. **Results:** The ZnO NPs exhibited potent antibacterial efficacy, achieving up to 99.999% killing against *Klebsiella pneumoniae*. When applied as an HPMC-ZnO coating, PP meshes and sutures demonstrated enhanced hydrophilicity, reducing water contact angles by ~41° and facilitating prevention of bacterial adhesion. The coated meshes inhibited bacterial attachment by 83% (*Escherichia coli*), 60% (*Pseudomonas aeruginosa*), 99.6% (*K. pneumoniae*), and 99% (*Staphylococcus aureus*). Similarly, coated sutures reduced adhesion by 67–96% across these strains. Long-term storage studies showed retained antibiofilm efficacy for up to six months. In vivo assessments indicated negligible systemic toxicity of ZnO NPs in murine models. **Conclusions:** Collectively, these findings highlight HPMC-ZnO NPs coatings as a safe, durable, and effective strategy to functionalize PP-based meshes and sutures, reducing the risk of surgical site infections and demonstrating the potential for broader biomedical applications.

## 1. Introduction

The increasing reliance on implantable medical devices has improved patient care, but not without risks [1]. The susceptibility of these medical devices to bacterial contamination leads to infections, irritation, and inflammation [2]. Once introduced into the body, the devices provide surface for bacteria to adhere, multiply, and produce extracellular polymeric substances, forming biofilms. Biofilm formed on the medical device is recalcitrant to antibiotic treatment and can evade the host immune system [3]. Bacterial colonization on the indwelling device can lead to functional impairment of the device, local tissue damage [4], and systemic dissemination, which contributes to high morbidity and mortality [5]. In this context, hernia, which is a medical condition characterized by the protrusion of an organ through the tissue or muscle lining, results in a visible bulge [6]. The majority of hernias require surgical interventions, with incisional hernia being significantly associated with post-operative complications and high disease burden. In surgical intervention, an incision is made at the herniated site, and the protruding tissue is repositioned and reinforced with a PP surgical mesh, followed by suturing of the operated site with PP material [7].

Globally, the prevalence of hernia increased by 36% between 1990 and 2019, from approximately 23.92 million to 32.53 million cases [8]. Post-operation complications of inguinal hernia range from ~2 to 20%, stemming from improper handling and contaminated mesh [9]. Open ventral hernia and laparoscopic hernia are linked to post-operative bacterial infection with a rate of approximately 10% to 3.6%, respectively [10]. The Centers for Disease Control and Prevention (CDC) report that surgical site infections (SSIs) are among the most common nosocomial infections, affecting around 17% of hospitalized patients [11]. Conventional sutures can harbor bacteria within their fibers, impairing wound healing at the incision site. *S. aureus* is responsible for up to 23% and 57.7% of SSIs associated with sutures and prosthetic mesh, respectively [10,12]. Around 26% of prosthetic mesh-associated infections are caused by Gram-negative bacteria, which mainly include *E. coli, Klebsiella spp*., and *P. aeruginosa* [13]. Different classes of antibiotics are commonly administered to manage post-operative complications. However, bacterial biofilm formation makes antibiotic treatment inefficient, with increased prevalence of drug resistance, particularly among nosocomial bacterial populations [14].

This situation highlights the need for good surface modifications to enhance the antibiofilm qualities of hernia meshes and surgical sutures. Recent attempts have been made to coat the surfaces of biomaterials by applying nanoparticles, particularly metal oxides like zinc oxide, silver oxide, and copper oxide nanoparticles, to prevent bacterial attachment and biofilm formation without compromising the mechanical properties of the device [15]. However, silver nanoparticles, though commonly used as antimicrobial coatings, have demonstrated limited efficacy, and the emergence of bacterial resistance against them underscores the need for more robust antimicrobial nanoparticles. Alternatively, ZnO NPs have shown potential outcomes due to their high surface area-to-volume ratio and charge density [16]. ZnO NPs exhibit multiple modes of action against microbial cells, such as contact killing by disrupting cellular integrity or by producing reactive oxygen species (ROS), which damage bacterial DNA and protein. Moreover, ZnO is recognized for its low toxicity to eukaryotic cells, reinforcing its suitability for biomedical applications [17,18,19,20,21,22]. Therefore, ZnO NP is a suitable antimicrobial agent that can be used for antibiofilm coatings for medical devices. While devices like urinary catheters have been functionalized using ZnO NP-based formulations, there are currently no reports detailing the development of ZnO NP-based antibacterial and antifouling coatings for surgical mesh and sutures. Polyethylene glycol (PEG), a synthetic polymer, is commonly used to functionalize nanoparticles on the surface of the biomaterial. However, in vivo studies have indicated that PEG metabolites may cause renal damage [23]. The adverse events associated with the material used to construct biomedical devices are often overlooked. Therefore, we chose HPMC, an FDA-approved polymer, which is a natural derivative of cellulose; it is safe, highly biocompatible, and less likely to cause adverse reactions, without compromising the device’s functional efficacy.

In our study, ZnO nanoparticles used for the surface coatings were synthesized using a mechanical-assisted thermal decomposition process [24]. Furthermore, a coating formulation was developed by amalgamating HPMC and ZnO NP. The coating formulation was applied to polypropylene hernia mesh and surgical sutures using a dip-coating technique [25]. SEM and EDAX analysis confirmed the deposition of the coating and transition to a more hydrophilic surface, implying an enhancement in the ability to inhibit microbial and protein adhesion. To ensure clinical relevance, we have used nosocomial isolates of clinically significant Gram-negative (*E. coli*, *P. aeruginosa*, *K. pneumoniae*) and Gram-positive (*S. aureus*) bacteria. During biofilm inhibition studies, we found that the coated surfaces can significantly inhibit bacterial adhesion compared to uncoated mesh and suture surfaces. Notably, the coatings retained their antibiofilm properties after six months of storage under dry and wet conditions, indicating high product stability. These findings indicate the potential of HPMC-ZnO NP coatings to reduce the risk of post-surgical complications associated with hernia operations, as well as the possibility of extending these coatings to other medical devices.

## 2. Results and Discussion

### 2.1. Characterization of ZnO Nanoparticles

#### 2.1.1. X-Ray Diffraction

**Figure 1:**
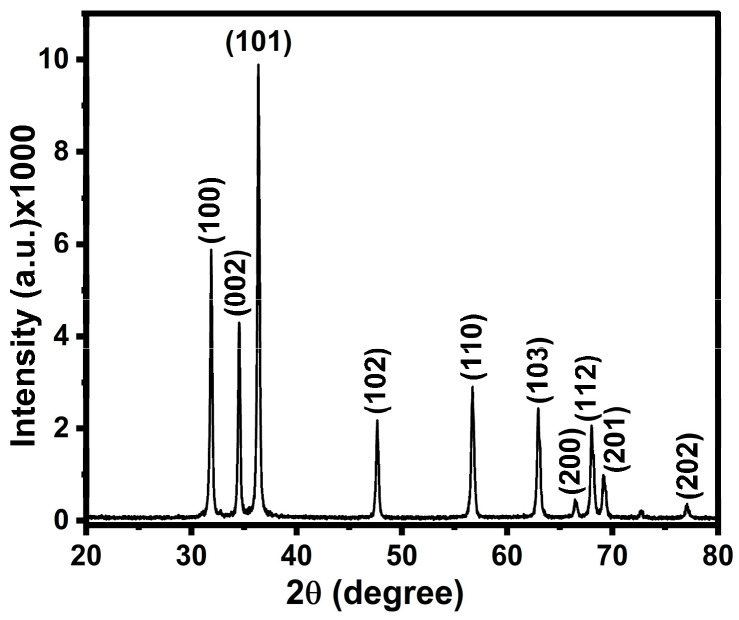
XRD pattern for the synthesized ZnO NPs shows characteristic peaks of ZnO.

The crystallinity and particle size of the ZnO NP were analyzed using XRD studies (Figure 1). In the XRD diffractogram, the peaks at scattering angle 2*θ*: 31.83°, 34.52°, 36.28°, 47.58°, 56.64°, 62.89°, 66.41°, 67.93°, 69.18°, and 77.01° correspond to the (100), (002), (101), (102), (110), (103), (200), (112), (201), and (202) planes, respectively, which are in agreement with JCPDS file no. 06-2151 [26,27,28]. The XRD diffractogram shows that the synthesized ZnO NPs have high purity and crystallinity. The different intensities of the other diffraction peaks are attributed to anisotropic growth in the various planes. The average particle size of ZnO was calculated by analyzing the dominant peak (101) using the Scherrer equation:

(1) $$D=\frac{0.9\lambda }{\beta cos\theta }$$

where *D* is the crystallite size of the particle, *λ* is the wavelength of the incident light, and *β* is the FWHM (full width at half maximum) of the peak located at 2θ [29]. The average crystal size of ZnO was calculated to be 73.72 nm. Furthermore, no diffraction peaks other than ZnO were detected, which indicates the high purity of ZnO NPs.

#### 2.1.2. FESEM and HR-TEM Show the Structure of Synthesized ZnO NPs

**Figure 2:**
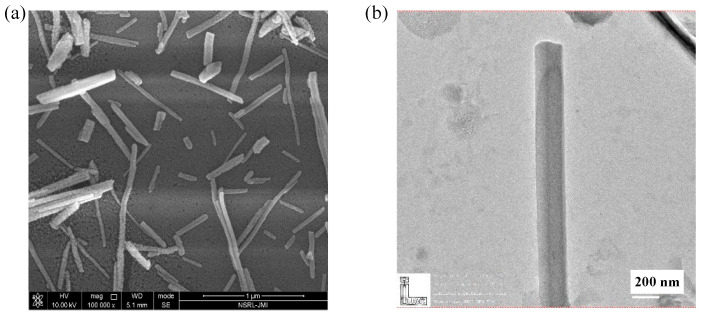
(**a**) FESEM showing rod-like structures of zinc oxide. (**b**) HR-TEM depicting the nanoscale dimensions of ZnO nanoparticles, confirming their crystallinity.

The morphology of the synthesized ZnO NP sample was investigated using FESEM and HR-TEM studies (Figure 2a). Annealing at high temperatures removed the impurities, resulting in well-grown nanoparticles, as shown in the FESEM image. Furthermore, the morphology of the ZnO nanoparticles was analyzed by HR-TEM at high magnification. The diameter of the ZnO nanoparticles calculated from the HR-TEM image is ~200 nm (Figure 2b). The crystal size (73.72 nm), as calculated from XRD, is less than the diameter (216.8 nm) of the rod as observed from HR-TEM, which implies that the nanoparticles are composed of several crystallites.

#### 2.1.3. FTIR Spectroscopy of ZnO Nanoparticles

**Figure 3:**
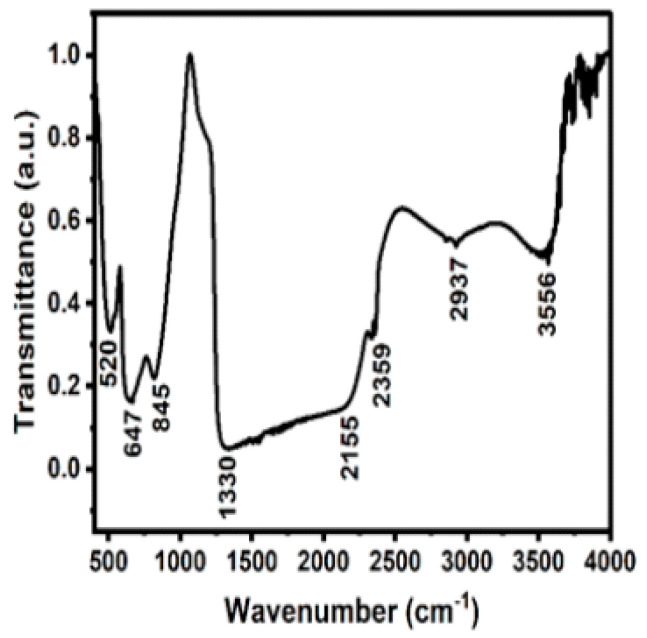
FTIR spectrum of ZnO nanoparticles.

The functional groups present on the surface of ZnO were studied using FTIR spectroscopy (Figure 3). The ZnO has absorption peaks in the range of 400–4000 cm^−1^. The absorption peaks at 520 and 647 cm^−1^ correspond to the metal–oxygen vibration mode, which is the main ZnO stretching vibration characteristic peak [30]. The absorption peak at 845 cm^−1^ is attributed to the formation of the tetrahedral coordination of Zn [31]. The peak located at 1330 cm^−1^ is attributed to the secondary alcohol in-plane bend vibration. The peaks located at 2155 and 2359 cm^−1^ correspond to the C-H stretching vibrations of the aromatic aldehyde. The peaks at 2937 and 3556 cm^−1^ correspond to the stretching vibration of the hydroxyl group, indicating the presence of a small amount of water molecules absorbed by the ZnO nanoparticles. The band at 1330 cm^−1^ can be attributed to C-O stretching vibrations of weakly adsorbed acetate originating from the zinc acetate used as a precursor in the synthesis of ZnO nanoparticles. The characteristic Zn-O stretching vibration observed below 600 cm^−1^ confirms the formation of ZnO nanorods. Additionally, the broad band around ~3556 cm^−1^ corresponds to surface hydroxyl (-OH) groups, which are intrinsic to ZnO nanostructures. The second-derivative spectrum (as shown in Supplementary Figure S1) reveals multiple overlapping -OH stretching components, suggesting the presence of hydroxyl groups in different surface coordination environments, such as isolated -OH, hydrogen-bonded -OH, and water molecules weakly bound to oxygen vacancies. The second-derivative spectrum confirms that these features are composed of multiple weak overlapping modes rather than a single intense impurity-related peak, indicating surface adsorption rather than bulk contamination. The band observed below around 520–647 cm^−1^ corresponds to the Zn-O stretching vibration, confirming the formation of ZnO nanoparticles.

#### 2.1.4. UV–Vis Spectral Analysis

**Figure 4:**
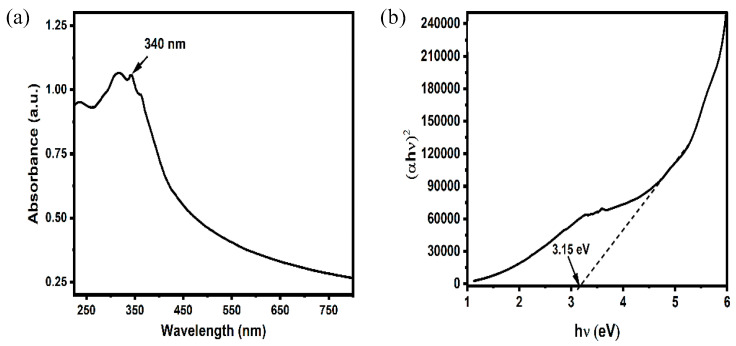
(**a**) UV–vis spectroscopy showing the characteristic absorption peak and (**b**) band gap of ZnO NP detected by Tauc’s plot.

UV–visible spectroscopy was utilized to investigate the optical properties of the ZnO NPs as shown in Figure 4a. The ZnO NPs have absorption in the range of 250–400 nm with a broad absorption peak at 340 nm, which is the characteristic peak of pure ZnO NP [32]. The absence of other peaks in the absorbance spectra indicates the purity of the synthesized ZnO NP. The band gap of the material was analysed using Tauc’s plot as shown in Figure 4b. The bandgap was calculated by extrapolating the line after the transition as shown by the dotted line, the intercept at x-axis gives the value of bandgap (Figure 4b). The band gap of the material was calculated by assuming the direct band gap transition and found to be 3.15 eV.

### 2.2. Antibacterial Activity of ZnO NP Against Clinical Strains of Gram-Positive and Gram-Negative Bacteria

ZnO, when reduced to nanoscale, can interact with bacterial surface and cause cell wall damage [30]. In suspension, ZnO NP can hinder bacterial growth via the release of Zn^2+^ ions, or by increasing levels of reactive oxygen species (ROS) [22,33]. The surface properties of ZnO NP are also crucial in their biocidal activity [34]. While ZnO NPs have a wide range of antibacterial effects, the precise mechanism behind their action remains uncertain. Interestingly, it is evident that the bactericidal action of ZnO NP involves multiple simultaneous mechanisms at once, potentially making nanoparticles a more effective treatment option than antibiotics, especially in light of the rapid emergence of antibiotic resistance [35].

To assess the antibacterial activity of ZnO NP, exponentially grown bacterial strains were treated with different concentrations (50, 100, 250, 500, and 750 µg/mL) of NPs for a period of 24 h. The effect of nanoparticles on bacterial growth kinetics was observed (Supplementary Figure S2), and the endpoint viable cell count was estimated. Untreated bacteria were used as control. A concentration-dependent inhibition of bacterial growth was observed for both Gram-positive as well as Gram-negative bacteria. Around 99.8% viable bacterial cell reduction was observed for *E. coli* ECU6 after treatment with ZnO NP at a concentration of 500 µg/mL. In case of *P. aeruginosa* PAO1, the viable cell reduction was around 87.5% at the highest treatment concentration. PAO1 produces a thick mucus-like substance, which might be responsible for the reduced antibacterial activity of the nanoparticle [36]. Maximum killing was observed for *K. pneumoniae* KPP1 with a reduction of 99.99% at a concentration of 250 µg/mL. The percentage reduction in viable cell count increased up to 99.9996% when the treatment concentration was increased to 750 µg/mL. A maximum killing around 99% was recorded for *S. aureus* SAW1, achieved at 500 µg/mL of ZnO NP concentration, which did not increase further even at higher concentrations (Supplementary Table S1). Among all the tested strains, ZnO NP showed maximum killing efficacy against *K. pneumoniae* with >5-log reduction in cell population at the highest tested concentration of ZnO NP. This indicated that the antibacterial action of ZnO NP is strain-specific and not Gram-specific.

### 2.3. In Vitro Antibiofilm Efficacy of ZnO NP Against Clinically Relevant Bacterial Strains

Biofilms play a crucial role in persistent and recurring bacterial infections. Antibiotics are often ineffective in eradicating biofilm-associated infections as biofilm harbors enhanced virulence factors [3]. The EPS layer prevents the complete adsorption of antimicrobial agents; hence, most treatments do not work against biofilm-associated infections or need a very high treatment concentration [37]. While antibiotics mainly target a single biosynthesis pathway in exponentially growing bacteria, ZnO NP not only targets key biosynthesis pathways but also induce ROS and exert direct contact-mediated killing, making them effective even against metabolically inactive bacterial cells, which are a major population in biofilms [38,39].

In this study, we examined the antibiofilm efficacy of ZnO NP by allowing the formation of the biofilm of clinically relevant strains in the presence of treatment at concentrations ranging from 250 to 750 µg/mL in 96-well microtiter plate. The results show a dose-dependent increase in antibiofilm efficacy against all tested strains (Supplementary Table S2). The treatment successfully reduced over 99% of biofilm viable cells of *E. coli* (ECU6) at the highest concentration of 750 µg/mL. At 500 µg/mL, it reduced around 99% of *K. pneumoniae* KPP1 biofilm, which further increased to 99.9% at 750 µg/mL of treatment concentration. In the case of *P. aeruginosa* PAO1, biofilm viable cells were reduced only by 64%, likely due to the enhanced EPS production. The alginate algC gene expression is shown to be upregulated in biofilm cells [40]. Over 90% reduction in biofilm viable cells was observed for *S. aureus* SAW1 at 750 µg/mL. ZnO NP effectively reduced the biofilm formation for all tested strains; hence, it can be used as a surface modification for medical devices to prevent medical device-associated infections.

### 2.4. SEM and EDAX Analysis of Coated Devices

**Figure 5:**
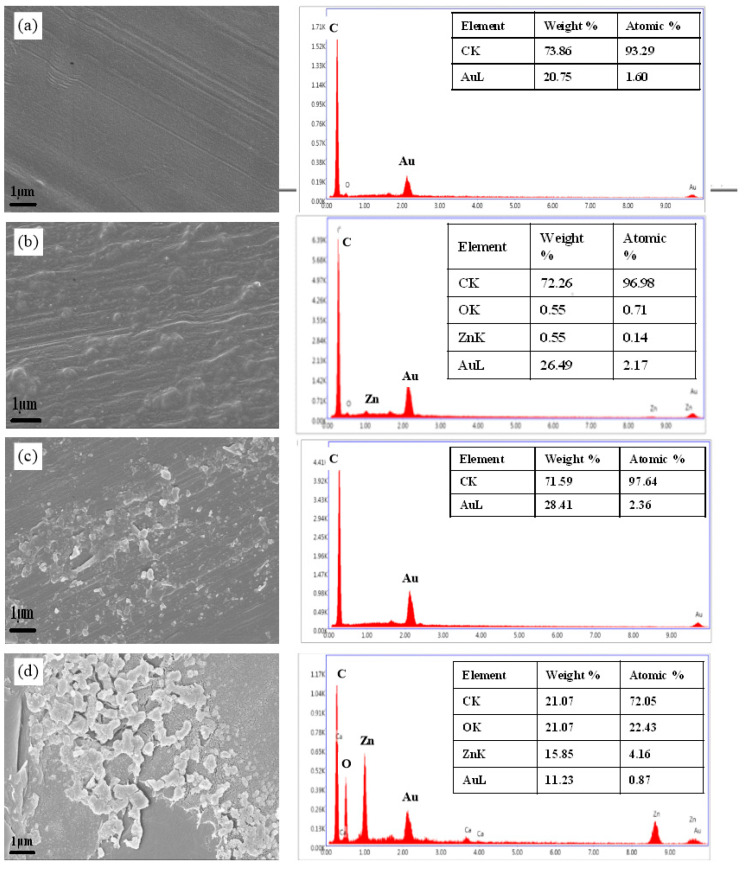
Characterization of HPMC-ZnO nanocomposite-coated polypropylene devices: SEM at a magnification of 10,000× and EDAX analysis shows surface morphology and elemental composition, respectively, of (**a**) uncoated mesh, which shows a smooth surface with peaks for carbon and gold (used for coating sample prior imaging). (**b**) Coated mesh shows a rough surface with peaks for carbon, gold, zinc, and oxygen atoms. (**c**) Uncoated suture shows a relatively smooth surface with an elemental composition of carbon and gold. (**d**) Coated suture shows a rough surface with peaks for carbon, gold, zinc, and oxygen atoms. Contact angle images of polypropylene mesh.

The SEM micrographs showed successful deposition of the coating solution on polypropylene mesh and sutures. The surface morphology of the control mesh appears smooth (Figure 5a), whereas the coated mesh exhibits a textured surface (Figure 5b), indicating the deposition of the coating formulation [41]. A similar observation is noted with uncoated (Figure 5c) and coated sutures (Figure 5d). Previous studies have shown HPMC to form a perfect matrix for the uniform dispersion of silver nanoparticles [42]. EDAX analysis of Zn and O ions in the coated mesh and suture was conducted, whereas the uncoated mesh and suture only have the background of carbon and gold. In a previous study from our lab, we successfully functionalized the catheter surface with phyto-assisted ZnO NPs [41]. The coating was highly effective in preventing bacterial colonization on the catheter surface. The growing concern about device-associated biofilm infection demands such innovative approaches to tackle secondary nosocomial bacterial infection. Furthermore, a rapid market translation of these safe and efficacious products could reduce the gap in basic research and clinical application. HPMC-based films incorporated with ZnO nanoparticles have also been developed for wound dressing application [43].

### 2.5. Change in Hydrophilicity of the Coated Polypropylene Mesh

**Figure 6:**
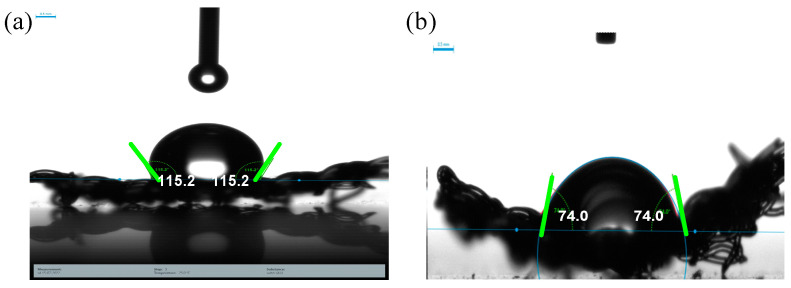
(**a**) Uncoated mesh showing a contact angle of 115.2°, indicating hydrophobic surface characteristics. (**b**) Coated mesh with reduced contact angle of 74°, demonstrating hydrophilicity.

Prior studies have stated that several important factors and parameters influence bacterial adhesion on a surface. One of the critical parameters is surface hydrophobicity. Therefore, we analyzed the change in surface charge by determining the water contact angle (CA) of HPMC-ZnO NP-coated and -uncoated mesh (Figure 6a,b). Uncoated mesh showed a CA of 115.72°, which means that the pre-coated surface was hydrophobic, and previously it has been shown that hydrophobic surfaces are prone to bacterial adhesion [44]. HPMC-ZnO NP coating significantly reduced the water contact angle to 74°, resulting in a hydrophilic surface. Several studies have reported the importance of wettability in bacterial adhesion on the surface. Liu et al., in their research, have shown the use of polymer PDMS in reducing the contact angle, which significantly decreased the bacterial adhesion for up to 2 days [45]. Hydrophilicity also plays a crucial role in surface biocompatibility and antifouling ability, as shown by Imbia et al. [46]. Surface contact angle measurement for sutures was not possible due to size restrictions. The suture sample dimensions with a length of 1cm and diameter of 0.5 mm were insufficient to provide a stable and measurable droplet interface.

### 2.6. HPMC-ZnO NP-Coated Devices Reduced In Vitro Bacterial Biofilm Formation

**Figure 7:**
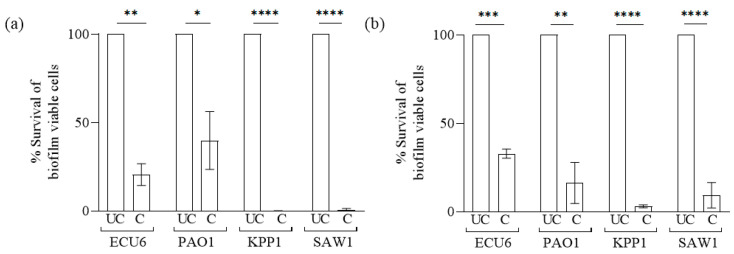
Percentage survival of biofilm cells on uncoated and coated (**a**) polypropylene mesh and (**b**) polypropylene suture. The X-axis represents the percentage biofilm viable cell count and the Y-axis denotes the bacterial strain ID. UC represents uncoated medical device and C represents the HPMC-ZnO-coated medical device. The uncoated and coated mesh and sutures were dipped in bacterial cell suspension and incubated for 3 h at 37 °C to allow cell adhesion, after which the devices were removed and washed with PBS; they were subsequently dipped in media and further incubated for 24 h. All experiments were performed in triplicate. Results are the mean ± standard deviations of the three individual experiments. Statistical analysis was performed by unpaired *t*-test with Welch’s correction using the GraphPad Prism software (version 8.0.1). Differences were considered significant at * *p* < 0.05, ** *p* < 0.005, *** *p* < 0.0005, and **** *p* < 0.0001.

ZnO has been used to develop antimicrobial and antifouling coatings for medical devices such as titanium implants [47], contact lenses [48], and orthopedic and dental implants [49]. We tested the antibiofilm efficacy of HPMC-ZnO NP-coated mesh and suture against *E. coli* (ECU6)*, K. pneumoniae* (KPP1)*, P. aeruginosa* (PAO1), and *S. aureus* (SAW1). The HPMC-ZnO NP-coated mesh showed only 20%, 39%, 0.05%, and 2% of ECU6, PAO1, KPP1, and SAW1 biofilm viable cell survival, respectively, compared to that of the uncoated mesh, which was designated as having 100% bacterial cell survival and used as the reference control (Figure 7a). Similarly, coated sutures showed only 32%, 16%, 3%, and 14% of ECU6, PAO1, KPP1, and SAW1 biofilm viable cell survival, respectively (Figure 7b). As observed previously, coated mesh exhibited increased hydrophilicity, indicated by a lower contact angle, which may have contributed to the observed reduction in bacterial adherence. The data is consistent with previous findings that hydrophilic surfaces tend to resist microbial attachment more effectively. Our results indicate, for the first time, the application of HPMC-ZnO NP as an antibiofilm coating formulation for hernia mesh and sutures. It can successfully reduce the risk of hernia-associated infections. Furthermore, in vivo studies are required to validate the interaction between host immunity, implanted devices, and bacteria.

### 2.7. Mode of Action and Durability of HPMC-ZnO NP-Coated Devices

ZnO NP coatings can prevent biofilm formation on the device surfaces by different modes of action [50]. Antibiofilm coatings can target bacteria via several routes: (1) Release-based coatings can release ions or nanoparticles that act as antimicrobial agents targeting bacterial cells adhered to the device’s surface and surrounding planktonic bacteria [51]. (2) For anti-adhesive coatings, the modified surface does not allow the bacteria to adhere to it due to the slip-and-slide phenomenon. (3) For antibacterial coatings, these coatings kill bacteria on adhesion. These coatings mainly cause cellular damage on contact [52]. We wanted to assess the antibiofilm mechanism of the HMPC-ZnO NP coating on hernia mesh and sutures. To do this, we dipped the uncoated and coated mesh in the growth media and evaluated the viable cells present in the growth media (free-floating bacteria) and those adhered to the medical devices, separately. In our study, we did not observe any change in bacterial viable counts in the growth media containing the coated mesh as compared to the control uncoated mesh (Supplementary Figure S3). However, there was a significant reduction in the bacteria adhered to the coated device compared to the uncoated device. We can estimate that HPMC-ZnO NP coatings either act as an anti-adhesive coating or kill bacteria on contact. Had there been release of ions or nanoparticles, it would have resulted in the reduction in viable cell count in the growth media in which the coated device was dipped. Since no such decrease in cells was observed, we ruled out the release of Zn^2+^ ions as the mechanism of action of our coatings. The inductively coupled plasma–mass spectrometry (ICP-MS) findings reveal the active concentration of zinc ions to be 390 ppb and 187 ppb in the leachate from coated mesh and sutures, respectively (Supplementary Table S3). Zinc ion concentrations lower than 330 ppm were previously shown to be non-toxic to microorganisms [53] and concentrations above this threshold show antibacterial activity particularly in *E. coli* [54]. The contribution of antibacterial activity from soluble zinc ions is only 15% [54]. Furthermore, as reported earlier, we also observed that upon contact, ZnO NPs led to the generation of intracellular ROS [55], resulting in the killing of bacteria. As shown in Supplementary Figure S4, ZnO nanoparticles induce strain-dependent but concentration-driven intracellular ROS generation, supporting oxidative stress as a major antibacterial mechanism. In *E. coli* and *K. pneumoniae,* exposure to 250 µg/mL of nanoparticle led to significant increase in ROS generation. On the contrary, the amount of intracellular ROS levels in *P. aeruginosa* PAO1 was quite low compared to all the tested strains, which may be attributed to robust oxidative stress defense mechanisms such as thick alginate production, which is a key ROS scavenger [56]. In case of *S. aureus*, it showed significant intracellular ROS induction even at 50 µg/mL of nanoparticle exposure, which further increased in a concentration-dependent manner. The elevated ROS response may indicate increased susceptibility of Gram-positive bacteria to ZnO NP-mediated oxidative stress, likely due to their cell wall architecture and lack of an outer cell membrane [57]. The ROS response for each strain correlates with the antibacterial efficacy of ZnO NPs shown in Supplementary Table S1.

**Figure 8:**
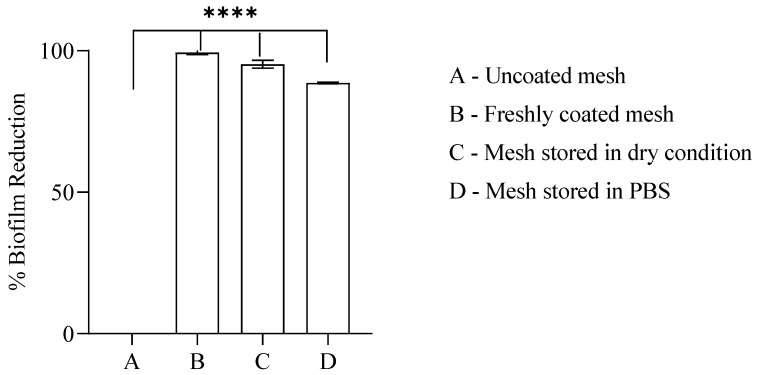
HPMC-ZnO NP coatings on surgical hernia mesh are stable up to 6 months of storage. The Y-axis represents the percentage *E. coli* (ECU6) biofilm viable cell reduction, and the X-axis denotes the different storage conditions. The sterile and stored mesh were dipped in bacterial cell suspension and incubated for 3 h at 37 °C to allow cell adhesion, after which the devices were removed and washed with PBS; they were subsequently dipped in media and further incubated for 24 h. Viable cell count was estimated by plating the harvested cells on TSB-agar plates. Results are the mean ± standard deviations of the mean of at least three independent experiments. Statistical analysis was performed by ordinary one-way ANOVA using the GraphPad Prism software (version 8.0.1). Differences were considered significant at **** p < 0.0001.

One of the challenges associated with nanoparticle-based coatings is their durability over a longer period of time. Few studies have discussed the lack of durability of commonly used antibacterial coating solutions such as PEG-based surface modifications [58], as well as layer-by-layer deposition of polyelectrolyte films [59]. We assessed the performance of the coatings by storing the coated devices under dry as well as wet conditions (in 1× PBS), while maintaining sterility for 6 months at room temperature. For our devices, we observed that after storage of coated devices in dry conditions for 6 months, the percentage biofilm inhibition reduced by just 4.36% compared to fresh coatings. On the other hand, the set of devices dipped in 1× PBS for the same number of days showed a reduction in the percentage biofilm inhibition by just 10.68% (Figure 8). However, devices kept under both conditions showed significant biofilm reduction compared to uncoated devices, indicating the stable long-term HMPC-ZnO NP coatings.

### 2.8. In Vivo Toxicology Assessment of ZnO NPs in Mice Model

**Figure 9:**
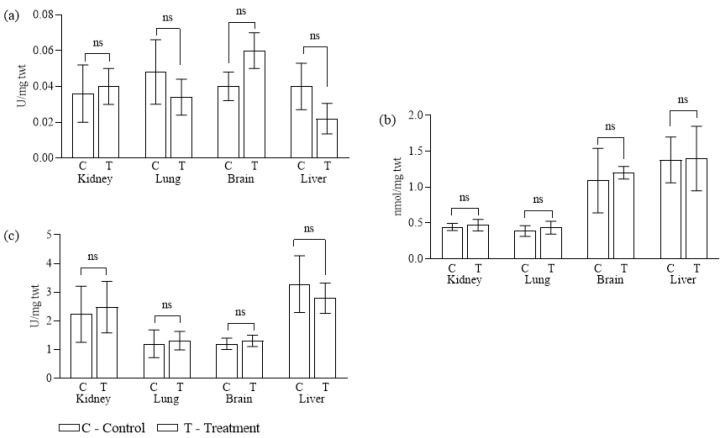
In vivo estimation of oxidative stress parameters in different tissues of mice injected with ZnO nanoparticles (T = treatment set) and without ZnO nanoparticles (C = control set). (**a**) GSH, (**b**) LPO, and (**c**) SOD. Mice (*n* = 3) were sacrificed after 7 days of treatment. Statistical analysis was performed by unpaired *t*-test with Welch’s correction using the GraphPad Prism software (version 8.0.1). Values that are not significantly different are represented with bars labeled as ns (non-significant).

Zinc oxide nanoparticles (ZnO NPs) are known to stimulate the generation of reactive oxygen species (ROS), which can disrupt intracellular metabolic processes and compromise the antioxidant defense system. The excess ROS produced under nanoparticle exposure can interact with vital biomolecules such as lipids, carbohydrates, proteins, and nucleic acids, leading to structural and functional damage [60]. The glutathione (GSH), lipid peroxidase (LPO), and superoxide dismutase (SOD) levels in the tissues of liver, kidney, brain, and lung in the control and ZnO NP-treated groups are shown in Figure 9 and further discussed below.

#### 2.8.1. Liver

Biochemical analysis of liver tissue from experimental animals revealed a significant reduction in glutathione (GSH) levels compared to control animals, suggesting oxidative stress and depletion of the tissue’s antioxidant reserves [61]. Interestingly, the level of lipid peroxidation (LPO) remained comparable between the experimental and control groups, indicating that ZnO NP exposure did not induce substantial damage to hepatocyte membranes. Superoxide dismutase (SOD) activity decreased by approximately 15% relative to the control. This decrease suggests that the applied ZnO NPs did not markedly enhance ROS formation in hepatocytes, thereby indicating their relatively high biocompatibility. These findings are noteworthy, as the liver is considered a primary target organ for ZnO NP accumulation and toxicity [62].

#### 2.8.2. Kidney

Comparative evaluation of kidney tissues between experimental and control groups showed no significant changes in either GSH or LPO levels, further supporting the absence of oxidative damage in renal cells. However, a slight increase of just over 10% in SOD activity was observed in the experimental group, likely reflecting an adaptive but limited antioxidant response to ZnO NP exposure.

#### 2.8.3. Brain

Given the susceptibility of neural tissues to oxidative imbalance, the activity of antioxidant enzymes was also assessed in brain tissue [63]. GSH activity showed a significant 50% increase in the experimental group, which may indicate the critical protective role of glutathione in counteracting nanoparticle-induced oxidative stress in the brain. SOD activity displayed only minimal change compared to controls, which could be explained by (i) lower accumulation of NPs in neural tissue, (ii) the predominance of alternative antioxidant systems, or (iii) limited permeability of the blood–brain barrier to SOD [61]. In contrast, LPO levels in brain tissue were significantly higher than in lung or kidney samples, suggesting enhanced susceptibility of neural cell membranes to peroxidative damage [64]. This observation may indicate active binding of ZnO NPs to neuronal membranes, thereby increasing their vulnerability.

#### 2.8.4. Lung

In lung tissue, GSH levels decreased by nearly 30% in mice receiving ZnO NPs compared to the control group, which is indicative of a substantial depletion of antioxidant reserves. Concurrently, LPO activity increased by approximately 11%, and SOD activity increased by around 9%, relative to the control groups. These combined alterations point to the moderate cytotoxic effect of ZnO NP exposure on pulmonary cells in rats.

## 3. Materials and Methods

### 3.1. Synthesis of Pure ZnO NPs

Pure ZnO nanoparticles were synthesized using a mechanical-assisted thermal decomposition process described in a previous study [28]. Briefly, mechanically ground zinc acetate dihydrate (Zn (CH_3_CO_2_)_2_.2H_2_O with 99.9% purity was finely ground and heated at 275 °C for 4 h at a controlled heating rate of 4 °C/min. The resulting material was washed thoroughly with Milli Q water to remove impurities and then dried at 100 °C for 8 h to yield ZnO nanoparticles.

### 3.2. Instrumentation for Characterization of ZnO Nanoparticles

The morphology of the synthesized ZnO NPs was analyzed using field emission scanning electron microscopy (FESEM) with a Quanta 3D FEG, (FEIs, Lausanne, Switzerland) and high-resolution transmission electron microscopy (HRTEM) with a JEOL/JEM-F200 (JOEL, Tokyo, Japan). The ZnO NPs were coated with gold before performing FESEM analysis. After dispersing in ethanol, the ZnO NPs was deposited on a carbon-coated copper TEM grid. The crystal structure of the ZnO NPs was analyzed using an X-ray diffractometer (XRD) from the Rigaku Smart Lab instrument (Tokyo, Japan). The band gap of the materials was calculated using Tuc’s plot from the absorbance spectrum. The absorbance spectrum of the ZnO NPs was studied using a UV–vis spectrometer (Agilent technologies Cary 100 series, Santa Clara, CA, USA). The bonds in the ZnO NP material were analyzed using Fourier transform infrared (FTIR) spectroscopy with the Bruker Tensor 37 FTIR-ATR spectrometer (Bruker, Billerica, MA, USA). The synthesized ZnO NPs were directly placed onto the ATR crystal (sensor), ensuring good physical contact, and the spectra were recorded. The FTIR spectra were recorded in the wavenumber range of 400–4000 cm^−1^.

### 3.3. Bacterial Strains and Growth Media

The bacterial strains used in the study were *P. aeruginosa* (PAO1) and clinical isolates of *E. coli* (ECU-6), *K. pneumoniae* (KPP1), and *S. aureus* (SAW1), which were obtained from Agartala Government Medical College (AGMC), Tripura, India. All the biological experiments on clinical isolates were conducted in a biosafety level 2 facility after the approval of protocols by the Institutional Biosafety Committee, Tripura University, vide certificate no. (TU/IBSC/2021/02) and the University of Delhi South Campus (IBKP UAC No. UNIRDARB3236), New Delhi, India. Gram-negative strains, i.e., PAO1, ECU-6, and KPP1, were grown in Luria–Bertani broth (LB, GM575, HiMedia Laboratories, Thane, India), and Gram-positive strain SAW1 was grown in Tryptic soy broth (GM011, HiMedia Laboratories, India) at 37 °C.

### 3.4. Antibacterial Efficacy of ZnO NPs Against Gram-Positive and Gram-Negative Bacteria

The antibacterial efficacy of ZnO NPs was assessed against both Gram-negative bacteria, *E. coli, P. aeruginosa,* and *K. pneumoniae,* and Gram-positive bacteria, *S. aureus.* The effect of increasing the concentration of nanoparticles on the growth of bacteria was determined by performing 24 h growth kinetics against each bacteria studied. Exponentially growing bacteria (OD_600_ 0.3–0.6) were treated with ZnO NPs at concentrations of 50, 100, 250, 500, and 750 µg/mL, and the growth kinetics were observed for 24 h at 37 °C with continuous shaking (Biotek Plate Reader, Agilent, USA). Untreated bacteria were kept as control. After incubation, serial dilution was performed, followed by spotting on agar plates; they were incubated overnight to obtain colony-forming units (CFUs) [65]. The percentage reduction in cell viability was determined by calculating the CFU/mL of treated bacteria compared to that of the control bacteria. All the experiments were performed in multiple replicates.

### 3.5. In Vitro Antibiofilm Efficacy of ZnO Nanoparticles Against Clinically Relevant Bacterial Strains

The antibiofilm efficacy of ZnO nanoparticles was determined using a 96-well microtiter plate assay [66]. Briefly, stationary phase bacteria of *E. coli*, *P. aeruginosa*, *K. pneumoniae,* and *S. aureus* were allowed to form biofilm in the presence and absence (control) of ZnO NP at the concentration of 250, 500, and 750 µg/mL. The plate was incubated at 37 °C for 24 h at static conditions. After 24 h, planktonic cells were washed thrice with phosphate saline buffer (PBS). CFU count was performed to determine the percentage of biofilm reduction. All the experiments were performed in multiple replicates.

### 3.6. Development of HPMC-ZnO NP Composite Coatings for Polypropylene Mesh and Sutures

Anti-adhesive coatings were developed using the dip-coating technique. An FDA-approved polymer HPMC was used as a binding agent for the nanoparticles on wearable medical devices. The coating solution consisted of 0.1% HPMC in an ethanol (81.25%)–water solution. This mixture added 0.005% *w*/*v* of ZnO nanoparticle synthesized at 275 °C. Evenly cut and washed polypropylene mesh (1 cm^2^) and polypropylene suture (1 cm by length) were dipped into the solution. The coating formulation with medical devices was kept on a magnetic stirrer for 3 h at 65 °C, 350 rpm. The devices were rinsed in ethanol and dried in hot air at 80 °C overnight. The devices were UV sterilized for 30 min before use.

### 3.7. SEM and EDAX Analysis of Deposition of ZnO NPs on the Surface of Coated Devices

To confirm the successful deposition of nanocomposite coating solution on devices, FE-SEM (Sigma-300, Carl Zeiss, Oberkochen, Germany) images were obtained for both coated and uncoated devices. The corresponding energy dispersive X-ray spectroscopy (EDAX) assessed the elemental chemical composition.

### 3.8. Surface Wettability Studies to Determine the Contact Angle

Contact angle analysis was performed using a DSA30E drop shape analyzer (KRÜSS GmbH, Hamburg, Germany), which was equipped with a precision camera and ADVANCE software 1.14 for droplet shape evaluation. A 10 μL droplet of ultrapure water (resistivity ≥ 18.2 MΩ·cm) was carefully applied to the film surface using a manual dosing mechanism. The contact angle was computed by fitting the droplet contour via the Young–Laplace equation. All measurements were conducted at room temperature (25 ± 1 °C), with three different locations per sample analyzed to account for spatial variability. The data are expressed as mean values ± standard deviation.

### 3.9. In Vitro Antibiofilm Efficacy of HPMC-ZnO NP-Coated Devices

The developed coatings on polypropylene mesh and sutures were tested against *E. coli* ECU6, *P. aeruginosa* PAO1, *K. pneumoniae* KPP1, and *S. aureus* SAW1. Coated devices were dipped in bacterial suspension at OD_600_ = 1 and incubated for 3 h at 37 °C under static conditions to promote initial adhesion. Uncoated devices served as control. After incubation, the devices were gently rinsed with 1X PBS three times to eliminate loosely attached planktonic bacterial cells. Finally, the mesh was transferred to fresh media and incubated at 37 °C for 24 h under static conditions to allow biofilm formation. After incubation, the devices were rinsed three times with PBS; this was followed by sonication for 1 min at 40 Khz (Ultrasonic bath sonicator, Labman, Chennai, India) and 1 min of vortexing to remove the adhered bacterial biofilm cells [67]. The serially diluted samples were spotted on LB agar or TSB agar plates to determine the percentage of viable bacterial cells (CFU/mL counts) in the biofilm that retained on coated devices compared to that on uncoated devices. All experiments were performed in triplicate with three biological replicates.

### 3.10. Mode of Action and Stability Assessment of Coated Devices

To understand the mode of action of our coatings, coated and uncoated devices were dipped in bacterial suspension and incubated for 24 h at 37 °C to allow the formation of *E. coli* (ECU6) biofilm. After 24 h, the devices were aseptically removed and washed to eliminate loosely adhered cells. The percentage of biofilm viable cell reduction for coated devices was calculated. Next, we calculated the concentration of bacteria (CFU/mL) in the cell suspension where the coated and uncoated devices were dipped.

ICP-MS analysis: Zinc ions released from ZnO NP-coated polypropylene mesh or suture were quantified by inductively coupled plasma–mass spectrometry (ICP-MS). The coated samples were immersed in bacterial growth media (Luria Broth prepared in Milli-Q water) for 7 days, to validate the release of Zn^2+^ ions. The leachate was ultracentrifuged (100,000× *g*, 4 °C for 1 h; Hitachi Himac (Tokyo, Japan), CS150FNX) to remove any residual ZnO nanoparticles; this was followed by filtration through a 0.22 µm PES syringe-driven filter (Minisart High Flow (Sartorius, Göttingen, Germany), Cat. No. 16532) membrane. The supernatant was acidified to 1% (*v*/*v*) trace-metal grade HNO 3 and diluted 100-fold prior to analysis. Measurements were performed in Triple Quadrupole ICP-MS (Agilent, Santa Clara, CA, USA, 8900) in He-collision mode, monitoring Zn at *m*/*z* 66, using external calibration with certified Zn standards and internal standard correction. Samples and procedural blanks were analyzed in triplicate, and concentrations were corrected for dilution and reported as µg·L^−1^ (ppb).

To measure intracellular ROS production, treated bacteria and untreated bacterial culture was centrifuged at 5000 rpm for 10 min, and the pellet was washed with PBS once. The pellet was resuspended in 1 mL of 1 mM 2′, 7′-Dichlorodihydrofluorescein diacetate (DCFH-DA) which is a non-fluorescent cell-permeable dye. The cellular esterases convert it to a non-fluorescent form which is DCFH. The intracellular ROS and peroxides oxidize DCFH to its highly fluorescent form dichlorofluorescein (DCF). The bacterial suspension in DCFH-DA is incubated at 37 °C for 30 min in dark conditions. Post incubation, the culture is centrifuged and the supernatant is discarded. The pellet is washed twice with PBS and finally resuspended in PBS. A total of 200 µL is transferred to a 96-well dark plate and fluorescence intensity is measured at an excitation wavelength of 488 nm and emission wavelength of 535 nm [68].

The stability of the coatings was tested in dry and wet conditions. We took a few coated devices from the same batch of coatings. Half were kept in sterile tubes; the rest were dipped in PBS and stored at room temperature for 6 months. Antibiofilm activity of freshly coated devices was recorded for comparison. After 6 months, devices from both dry and wet conditions were subjected to antibiofilm analysis, and their viable cell reduction was compared to the biological activity shown by freshly coated devices recorded previously. The uncoated device was kept as control.

### 3.11. In Vivo Toxicology Assessment of ZnO NP in Mice Model

Male BALB/c mice (6–8 weeks old) were procured from the Zydus Research Center (Ahmedabad, India). Animals were maintained under standard laboratory conditions (12 h light/dark cycle, a controlled temperature of 22 ± 1 °C, and relative humidity of 55 ± 1%) with ad libitum access to autoclaved water and standard rodent chow. All experimental procedures were reviewed and approved by the Institutional Animal Ethics Committee of National Institute of Occupational Health (IAEC/NIOH/2018-19/21/02/M), on 25 April 2019. They were also performed in accordance with CPCSEA guidelines (Prevention of Cruelty to Animals Act 1960, India) and reported following the ARRIVE 2.0 guidelines.

Prior to the initiation of experiments, animals were acclimatized for one week. Following acclimatization, mice were randomly assigned to experimental groups (n = 3 per group). Treatment groups received a subcutaneous injection of zinc oxide nanoparticles (50 µg per mouse) suspended in 100 µL sterile 1× phosphate-buffered saline (PBS), whereas controls received 100 µL PBS alone. Body weights were recorded daily to monitor systemic responses.

After seven days of treatment, mice were euthanized, and major organs including the brain, lungs, liver, and kidneys were harvested. The tissues were processed to evaluate oxidative stress and antioxidant parameters, specifically glutathione (GSH) levels, lipid peroxidation (LPO), and superoxide dismutase (SOD) activity, to assess cellular and pathological responses to the administered nanoparticles.

### 3.12. Statistical Analysis

All experiments were performed in triplicate unless stated otherwise. The GraphPad Prism software was used for statistical analysis. Full details of all statistical tests can be found in the main text and the corresponding result can be found in figure legends.

## 4. Conclusions

Surface coatings for hernia kits, consisting of polypropylene mesh and sutures, must be able to resist bacterial infection as well as inhibit blood protein adhesion. The importance of such therapeutic strategies has been acknowledged as essential in preventing implant-associated infections. In this study, we demonstrated that the ZnO nanoparticles showed a concentration-dependent antibacterial efficacy against clinically relevant Gram-positive and Gram-negative bacteria. The synthesized nanoparticles showed rod-like morphology with a high aspect ratio of 18.75. Furthermore, we developed a one-step composite coating solution with a combination of polymer HPMC and ZnO NPs for polypropylene hernia mesh and sutures. HPMC acts as an adhesive layer to promote even coatings of ZnO nanoparticles on the surface of polypropylene mesh and sutures. Moreover, the coating improved the antibiofilm efficacy of surgical hernia mesh and sutures against all the tested bacteria. Furthermore, we evaluated the mode of action of our coatings, concluding that HPMC-ZnO NPs coatings were not release-based but depended on contact killing. The HPMC-ZnO NP coatings also showed long-term stability over the period of six months under wet and dry conditions. Importantly, biocompatibility was demonstrated by the fact that the ZnO NPs had non-significant toxicity in the in vivo model. Overall, the HPMC-ZnO NP nanocomposite coating solution has the potential to be translated into various multi-functional coating applications for various biomedical implants due to its ease of development and broad-spectrum antibacterial efficacy, which promises anti-adhesive property and biocompatibility.

The antibiofilm efficacy of the coated devices was evaluated primarily under in vitro static conditions, which may not fully replicate the complex physiological environment encountered in vivo, which includes mechanical stress and host immune interactions. The present study is focused on a limited set of clinically relevant bacterial strains. Expanding future investigations to include polymicrobial biofilms, antibiotic-resistant strains, and fungi, which are commonly associated with surgical site infections, would further strengthen the translational relevance of the coatings.

## Data Availability

The original contributions presented in the study are included in the article/Supplementary Material; further inquiries can be directed to the corresponding author.

## Supplementary Materials

The following supporting information can be downloaded at https://www.mdpi.com/article/10.3390/ph19010055/s1; Figure S1: The second-derivative spectrum showing multiple overlapping -OH stretching components; Figure S2: Effect of ZnO NP on the growth of (a) *E. coli* ECU6, (b) *P. aeruginosa* PAO1, (c) *K. pneumoniae* KPP1, (d) *S. aureus* SAW1 at concentrations of 50 μg/ml, 100 μg/ml, 250 μg/ml, 500μg/ml and 750μg/ml. Untreated bacteria is kept as the Control; Figure S3: Mode of action of ZnO-HPMC nanocomposite coatings. All experiments were done in triplicate. Results are the mean ± standard deviations of three individual experiments. Statistical analysis was done by unpaired *t*-test with Welch’s correction using GraphPad Prism software (version 8.0.1). Differences were considered significant at *** p* < 0.005; Figure S4: Intracellular Reactive oxygen species (ROS) generation in (**a**) *E. coli* ECU6 (**b**) *P. aeruginosa* PAO1 (**c**) *K. pneumoniae* KPP1 (**d**) *S. aureus* SAW. Data are expressed as mean ± standard deviation (SD) from multiple independent experiments. Statistical analysis was done by unpaired *t*-test and ordinary One-way ANOVA using GraphPad Prism software version 8.0.1. Differences were considered significant at *p* < 0.01 **, *p* < 0.001 ***, *p* < 0.0001 ****, ns represents non-significant; Table S1: Percentage reduction of viable bacterial cells after exponentially growing bacteria was treated with ZnO NP at different concentrations for a time period of 24 h; Table S2: Percentage reduction of biofilm viable cels after treatment with ZnO NP at different concentrations. Table S3: ICP-MS analysis for estimating the concentrations of Zn^2+^ ions (μg/L) released from coated mesh immersed in bacterial growth media.

## Abbreviations

BAI	Biomedical Device-Associated Infection
PP	Polypropylene
HPMC	Hydroxypropyl Methylcellulose
ZnO	Zinc Oxide
NP	Nanoparticle
FE-SEM	Field Emission Scanning Electron Microscope
EDAX	Energy Dispersive X-ray Spectroscopy
XRD	X-Ray Diffraction
FWHM	Full Width at Half Maximum
HRTEM	High-Resolution Transmission Electron Microscopy
FTIR	Fourier Transform Infrared Spectroscopy
EPS	Extracellular Polymeric Substance
PBS	Phosphate Saline Buffer
OD	Optical Density
