# Supplementary material for: HPMC-ZnO Nanorods Enhance Hydrophilicity and Contact-Killing Activity on Polypropylene Meshes and Sutures
Source: Pharmaceuticals (Basel). 2025 Dec 26;19(1):55. doi: 10.3390/ph19010055 (PMC12844732; doi:10.3390/ph19010055)
Supplement: Supplementary file 1 [file pharmaceuticals-19-00055-s001.zip › pharmaceuticals-4024278-supplementary.pdf]

Supplementary information

HPMC-ZnO Nanorods Enhance Hydrophilicity and Contact-Killing Activity on Polypropylene Meshes and Sutures

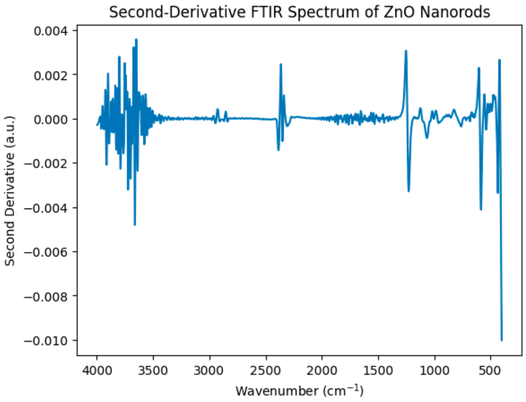

Supplementary Figure S1: The second-derivative spectrum showing multiple overlapping -OH stretching components.

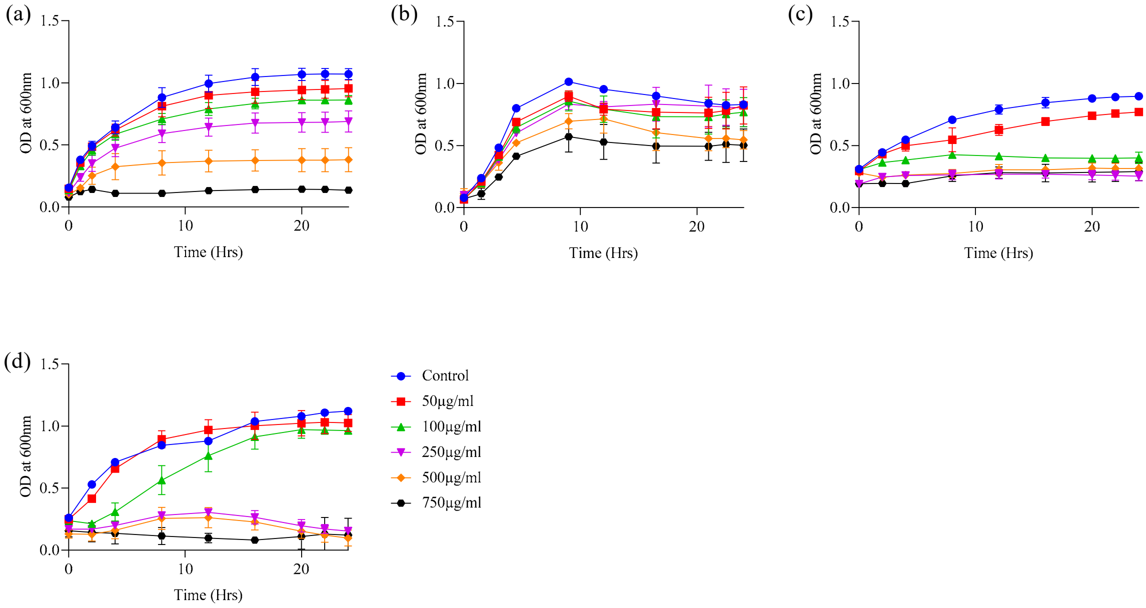

Supplementary Figure S2: Effect of ZnO NP on the growth of (a) *E. coli* ECU6, (b) *P. aeruginosa* PAO1, (c) *K. pneumoniae* KPP1, (d) *S. aureus* SAW1 at concentrations of 50µg/ml, 100µg/ml, 250µg/ml, 500µg/ml and 750µg/ml. Untreated bacteria is kept as the Control.

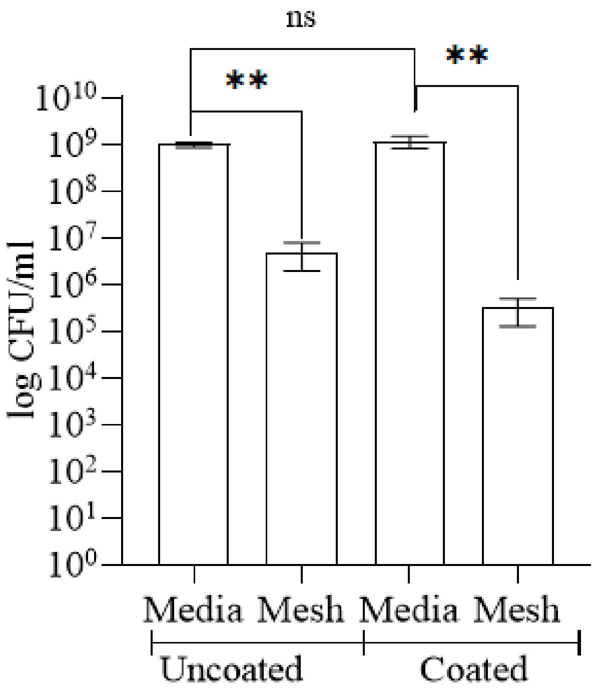

Supplementary Figure S3: Mode of action of ZnO-HPMC nanocomposite coatings. All experiments were done in triplicate. Results are the mean ± standard deviations of three individual experiments. Statistical analysis was done by unpaired t-test with Welch's correction using GraphPad Prism software (version 8.0.1). Differences were considered significant at ***P < 0.005.*

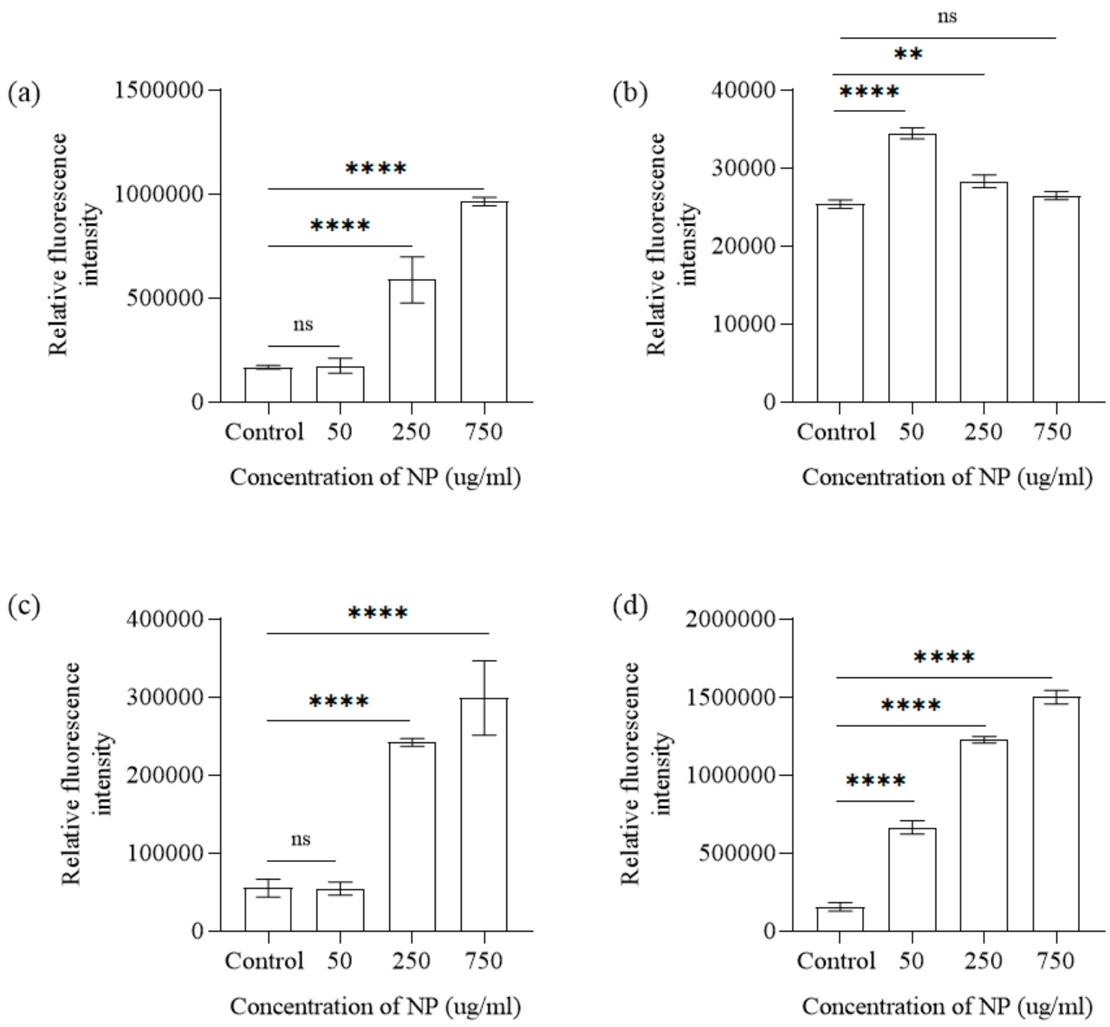

Supplementary Figure S4: Intracellular Reactive oxygen species (ROS) generation in **(a)** *E. coli* ECU6 **(b)** *P. aeruginosa* PAO1 **(c)** *K. pneumoniae* KPP1 **(d)** *S. aureus* SAW. Data are expressed as mean ± standard deviation (SD) from multiple independent experiments. Statistical analysis was done by unpaired t-test and ordinary One-way ANOVA using GraphPad Prism software version 8.0.1. Differences were considered significant at *p<0.05, p<0.001**, p <0.0001****, ns* represents non-significant.

Supplementary Table S1: Percentage reduction of viable bacterial cells after exponentially growing bacteria was treated with ZnO NP at different concentrations for a time period of 24 hours.

Strain Id.	50µg/ml	100µg/ml	250µg/ml	500µg/ml	750µg/ml
ECU6	26%	37.8%	75.2%	98.8%	99.8%
PAO1	24%	46%	72%	85%	87.5%
KPP1	43%	84%	99.99%	99.998%	99.9996%
SAW1	33%	59%	95.4%	99.2%	99.1%

Supplementary Table S2. Percentage reduction of biofilm viable cels after treatment with ZnO NP at different concentrations.

Strain Id.	250µg/ml	500µg/ml	750µg/ml
ECU6	34.8%	93.8%	99.67%
PAO1	33%	56.75%	64.25%
KPP1	55.6%	99.4%	99.96%
SAW1	0%	87.87%	98.37%

Supplementary Table S3. ICP-MS analysis for estimating the concentrations of Zn^{2+} ions (µg/L) released from coated mesh immersed in bacterial growth media.

Sample Description	$[Zn^{2+}]$ µg/L	ppm
ZnO NPs coated mesh	390	0.39
ZnO NPs coated sutures	187	0.18
